# Evaluation of quality of life and psychological aspects of Parkinson's disease patients who participate in a support group

**DOI:** 10.1590/1980-57642015DN93000013

**Published:** 2015

**Authors:** Nathalie Ribeiro Artigas, Vera Lúcia Widniczck Striebel, Arlete Hilbig, Carlos Roberto de Mello Rieder

**Affiliations:** 1 Physiotherapist, Postgraduate Program in Rehabilitation Sciences, Universidade Federal de Ciências da Saúde de Porto Alegre RS, Brazil.; 2 Physiotherapy School, Centro Universitário Metodista do IPA, Porto Alegre RS, Brazil.; 3 Neurology, Clinical Medicine Department - Universidade Federal de Ciências da Saúde de Porto Alegre RS, Brazil.; 4 Neurology, Clinical Medicine Department, Universidade Federal de Ciências da Saúde de Porto Alegre RS, Brazil; Postgraduate Program in Medical Science, Universidade Federal de Ciências da Saúde de Porto Alegre RS, Brazil.

**Keywords:** Parkinson's disease, quality of life, anxiety, depression

## Abstract

Parkinson's disease (PD) is a neurodegenerative disorder that can dramatically impair patient quality of life (QoL).

**Objective:**

To analyze the QoL, motor capacity, depression, anxiety and social phobia of individuals who attended a patient support group (PSG) compared to non-participants.

**Methods:**

A cross-sectional study was performed. The sample consisted of 20 individuals with PD who attended a PSG and another 20 PD patients who did not attend a support group for PD patients, serving as the control group (nPSG). All patients answered questionnaires on motor capacity (UPDRS), QoL (Parkinson's Disease Questionnaire- PDQ-39), depression (Beck Depression Inventory), anxiety (Beck Anxiety Inventory) and social phobia (Liebowitz Social Anxiety Scale). To determine data distribution, the Shapiro-Wilk test was performed. For comparison of means, Student's t-test was applied. In cases of asymmetry, the Mann-Whitney test was employed. To assess the association between the scales, Pearson's correlation coefficient (symmetric distribution) and Spearman's coefficient (asymmetric distribution) were applied. For the association between qualitative variables, Pearson's Chi-squared test was performed. A significance level of 5% (p≤0.05) was adopted.

**Results:**

Individuals in the PSG had a significantly better QoL (p=0.002), and lower depression (p=0.026), anxiety (p<0.001) and social phobia (p=0.01) scores compared to the nPSG.

**Conclusion:**

The participation of PD patients in social activities such as support groups is associated with better QoL and fewer symptoms of depression, anxiety and social phobia.

## Abstract (pt)

A doença de Parkinson (DP) é uma doença neurodegenerativa que pode prejudicar a qualidade de vida (QV) do paciente.

**Objetivo:**

Analisar a qualidade de vida, capacidade motora, depressão, ansiedade e fobia social dos indivíduos que frequentavam um grupo de apoio a pacientes (PSG) em comparação com aqueles que não frequentavam.

**Métodos:**

Trata-se de um estudo transversal. Amostra composta por 20 indivíduos com Parkinson que participavam de uma PSG e outros 20 pacientes com DP que não participavam de qualquer grupo de apoio para pacientes com DP, denominado de grupo de controle (nPSG). Os participantes responderam questionários sobre capacidade motora (UPDRS), QV (PDQ-39), depressão (Inventário de depressão de Beck), ansiedade (Inventário de ansiedade de Beck) e fobia social (Escala de Ansiedade Social de Liebowitz). Determinando a distribuição dos dados, foi realizado o teste de Shapiro-Wilk. Comparando médias, teste t de Student foi aplicado. Em assimetria, foi realizado o teste de Mann-Whitney. Avaliando a associação entre as escalas, o coeficiente de correlação de Pearson (distribuição simétrica) e o coeficiente de Spearman (distribuição assimétrica) foram aplicados. Para a associação entre as variáveis qualitativas, foi realizado o teste qui-quadrado de Pearson. Adotado o nível de significância de 5% (p≤0,05).

**Resultados:**

Os indivíduos do PSG apresentaram uma qualidade de vida significativamente melhor (p=0,002), menos depressão (p=0,026), ansiedade (p<0,001) e fobia social (p=0,01) quando comparado com o nPSG.

**Conclusão:**

A participação de pacientes com DP em atividades sociais como os grupos de apoio está associado a melhor qualidade de vida e menor presença de sintomas de depressão, ansiedade e fobia social.

## INTRODUCTION

Parkinson's disease (PD) is the second-most-common chronic neurodegenerative disease, affecting 3.3% of individuals over the age of 65.1 The disease causes several motor and non-motor symptoms that may affect the quality of life (QoL) of patients and caregivers.

Besides the classical motor symptoms (rigidity, bradykinesia and tremor), anxiety, sleep disorders, social isolation, memory loss and depression are factors that can reduce the QoL in this group.^2-4^ Improvement in QoL requires symptom relief, where it is crucial for PD patients to be followed by a multidisciplinary care team (nurses, neurologist, occupational therapist, physiotherapist, speech-language pathologists, among others), with individual or group treatment, promoting potential improvements in patient functional capacity, well-being and QoL.^3,5,6^

Many patients find it hard to cope with PD. It may be hard for them to ask their doctor questions or talk about their problems with family or friends. Furthermore, the frequency of neurological appointments may be lower than needed, especially in low-income communities. Patient support groups (PSG) may be helpful for PD patients. Meetings with other PD sufferers may be a source of encouragement for many patients and provide an opportunity to discuss experiences and feelings, and to share solutions to common problems.^7^

To the best of our knowledge, there are no studies evaluating the impact of Parkinson's PSG on patient QoL in the Brazilian population. The main objective of this study was to assess the QoL of PD patients who attend a PSG. The secondary objectives were to compare cognitive and motor capacities, prevalence of depression, anxiety and social phobia between patients who attended a PSG and those who did not with the aim of identifying differences in psychological aspects among individuals that have social support in their everyday lives.

## METHODS

**Study design.** The present cross-sectional design study was performed in 40 patients followed by neurologists or a PSG and conducted in Porto Alegre, RS - Brazil, between 2011 and 2012.

**Subjects.** For this study, the selected participants were divided into two groups. The first, the Patient Support Group (PSG) comprised 20 individuals with PD who attended a PSG; the second, the control group (nPSG) comprised 20 PD patients who did not attend a support group for PD patients. All patients were accompanied by a neurologist.

PSG participants were invited to take part in the study by contacting all patients who attended a PSG called APARS (Parkinson's Association of Rio Grande do Sul). The nPSG patients were invited after being referred by neurologists who agreed to collaborate with the study, where these individuals had no contact with a PSG.

The inclusion criteria for both groups were: diagnosis of PD by a neurologist, aged 45 years or older, no dementia according to clinical criteria, and a score above 24 on the Mini–Mental State Examination (MMSE).^8,9^

The exclusion criteria were: individuals who did not participate in the activities proposed by the PSG at least twice a month (only for the PSG group) and individuals diagnosed with other neurologic diseases.

Initially, all individuals who attended the APARS and met the inclusion criteria were included, totaling 24 individuals. However, 4 individuals refused to participate in the study, giving a final total of 20 participants in the PSG group. After describing the study to the participants, a meeting was scheduled, according to the availability of each participant to sign the Informed Consent Form. The assessment instruments were then applied.

**Assessment procedures.** All ratings were performed one hour after use of antiparkinsonian medication (levodopa or levodopa equivalents) with patients during an ON period. The researcher who conducted the assessment of participants was blinded to the information on which group each patient belonged to.

Data regarding age, gender and time since diagnosis were collected. Disease severity was measured by the Hoehn and Yahr Scale^10^ and the motor section of the Unified Parkinson's Disease Rating Scale (UPDRS).^11^

To assess individual QoL, the Parkinson's Disease Questionnaire (PDQ-39) was applied.^12^ This is a 39-item scale divided into 8 categories: mobility, activities of daily living, emotional well-being, stigma, social support, cognition, communication and bodily discomfort. Total score ranges from 0 (no impairments) to 100 (maximum level of impairment); i.e., a low score indicates a better self-perceived QoL by the individual.

Symptoms of depression were assessed using the Beck Depression Inventory (BDI), composed of 21 questions. Items 1 to 13 evaluate psychological symptoms, whereas items 14 to 21 evaluate physical (somatic) symptoms. Scores of up to 9 indicate no depression or minimal depression; from 10 to 18, mild to moderate depression; from 19 to 29, moderate to severe depression; and from 30 to 63, severe depression.^13^

The Beck Anxiety Inventory (BAI) was applied to assess anxiety symptoms of the subjects. This is a 21-item instrument (each item scored from 0 to 3), and the sum of the scores give a result from 0 to 63. Total scores of up to 7 indicate a minimum level of anxiety; from 8 to 15, mild anxiety; from 16 to 25, moderate anxiety; and from 26 to 63, severe anxiety.^14^

The last instrument applied in the interview was the Liebowitz Social Anxiety Scale (LSAS), comprising 24 items, assessed in two categories: fear and avoidance. The sum of the scores result in three categories of social phobia: mild (51 or below), moderate (from 52 to 81) and severe (82 or above).^15^

**Statistical analysis.** For the statistical analysis, the continuous variables: age, cognitive capacity, motor capacity, QoL and depression were expressed as mean and standard deviation (symmetric distribution), whereas the variables: disease duration, anxiety and social phobia were expressed as median and interquartile range (asymmetric distribution). The qualitative variables gender and disease stage were expressed as absolute and relative frequencies. The Shapiro-Wilk test was performed to determine data distribution. For comparison of means, Student's t-test was applied. In cases of asymmetry, the Mann-Whitney test was employed. To assess the association between the scales, Pearson's correlation coefficient (symmetric distribution) and Spearman's coefficient (asymmetric distribution) were applied. For the association between qualitative variables, the Pearson's Chi-squared test was performed. A significance level of 5% (p≤0.05) was adopted and all tests were performed using SPSS (Statistical Package for the Social Sciences) software, version 17.0.

**Ethical aspects.** This study was approved by the Ethics Committee of the *Centro Universitário Metodista do IPA*, Porto Alegre, RS, Brazil, process number 367/2010 and was performed according to the Code of Ethics of the World Medical Association (Declaration of Helsinki).

## RESULTS

The sample consisted of 40 individuals, split into two groups of 20 participants each. Sample characteristics are given in Table 1.

**Table 1:** Sample characteristics.

Variables		PSG (n=20)	nPSG (n=20)	p
Age	Age	64.3 SD 8.3	66.5 SD 6.5	0.346
Gender – n (%)	Male	13 (65)	10 (50)	0.552
	Female	7 (35)	10 (50)	
Duration of disease - median (P25- P75)		6.75 (4.2-12)	9 (4-12)	0.659
Hoehn & Yahr Staging - n (%)	1	0 (0.0)	1 (5.0)	0.532
	1.5	7 (35.0)	3 (15.0)	
	2	1 (5.0)	2 (10.0)	
	2.5	5 (25,0)	4 (20.0)	
	3	6 (30.0)	7 (35.0)	
	4	1 (5.0)	3 (15.0)	

PSG: patient support group; nPSG: control group; n: sample size; SD: standard deviation; %: percentage; P25: 25^th^ percentile; P75: 75^th^ percentile: p: significance level for comparison between groups.

Both groups were homogeneous for performance on the MMSE (p=0.062) and UPDRS (p=0.576), as described in Table 2.

**Table 2:** Comparison between groups.

Variables	PSG (n=20)	nPSG (n=20)	p
Cognitive capacity	28.0 SD 1.6	26.62.8	0.062
Motor capacity	24.0 SD 6.4	25.3 SD 8.6	0.576
Quality of life	30.3 SD 9.7	42.1 SD 12.1	0.002
Depression	15.5 SD 6.8	25.2 SD 11.6	0.026
Anxiety – median (P25–P75)	10 (6–16)	23.5 (16.5–34.3)	<0.001
Social phobia – median (P25– P75)	34 (11.5–51.3)	69 (48.3–93)	0.001

PSG: patient support group; nPSG: control group; n: sample size; %: percentage; SD: standard deviation; P25: 25^th^ percentile; P75: 75^th^ percentile.

Table 2 displays the comparison between PSG and nPSG groups for QoL, depression, anxiety and social phobia. PSG group participants had a significantly better QoL and lower depression, anxiety and social phobia scores compared to nPSG group individuals.

**Figure 1:**
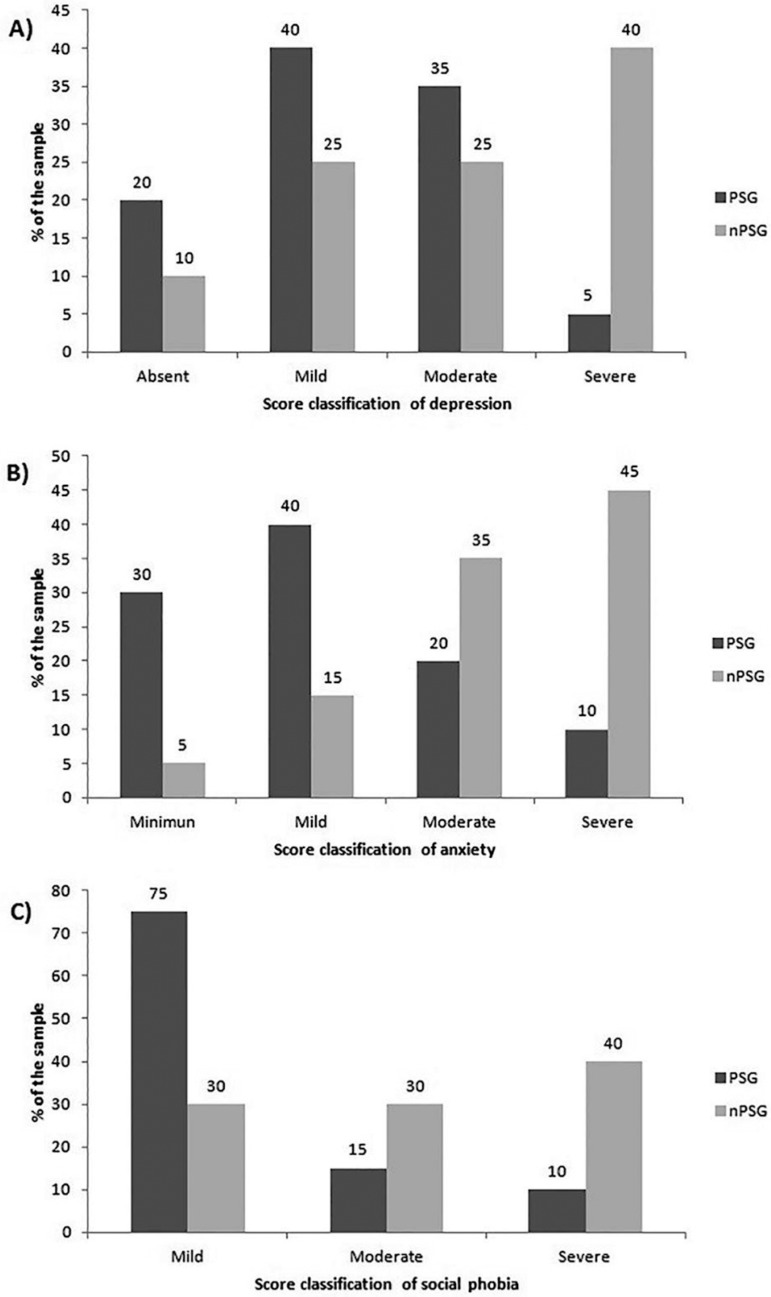
Classification of individuals according to depression, anxiety and social phobia scores.

Figure 1A illustrates the sample distribution regarding severity of depression. A total of 40% of PSG participants exhibited mild depression scores while 40% of the control group subjects had severe depression scores. Figure 1B shows that for anxiety levels, 40% of PSG individuals had mild anxiety scores, whereas 45% of nPSG individuals exhibited severe anxiety.

In the PSG group, 75% of the individuals showed mild social phobia, whereas in the nPSG, 40% had severe social phobia (Figure 1C).

Table 3 describes correlations between the variables assessed. Comparisons were performed on the whole sample (n=40). There was a statistically significant moderate correlation between motor capacity and the variables QoL (r=0.428 and p=0.006) and depression (r=0.427 and p=0.006). A correlation was also evident between QoL and depression (r=0.448 and p=0.004) and anxiety (r=0.634 and p≤0.083).

**Table 3:** Correlations between variables.

	Motor capacity			Quality of life	
	Correlation coefficient*	p		Correlation coefficient*	p
Quality of life	0.428	0.006		–	–
Depression	0.427	0.006		0.448	0.004
Anxiety	0.297	0.063		0.634	<0.001
Social phobia	0.298	0.062		0.277	0.083

* Pearson’s correlation for quality of life and depression, and Spearman’s for anxiety and social phobia.

## DISCUSSION

Our results suggest that the participation of PD patients in PSG is associated with better QoL, fewer depression and anxiety symptoms and less social phobia. In this study, patient participants or non-participants in PSG were evaluated. There was homogeneity between groups regarding cognitive and motor symptoms.

Currently, health care practice focuses on enhancing QoL and seeking strategies to promote a healthier life, especially for those patients with chronic diseases.^4^ In order to obtain a better relationship with the world and, consequently, an improvement in QoL of PD patients, it is fundamental to assess which factors are central to building a socializing process that may motivate these individuals thereby improving adherence to treatment of the disease and its consequences. In order to promote these supportive measures and education of PD patients, the Parkinson's Association of Rio Grande do Sul (APARS) was founded in September, 2000. The participants in the PSG group were members of this association.

The results demonstrated that the PSG patients had better QoL compared to patients who did not participate in the group. This result suggests that joining a PSG and receiving more information about the illness, as well as having contact with other patients with similar complaints can help patients realize they are not the only ones suffering from the condition. This provides an opportunity for these individuals to share their anxieties and to feel capable of overcoming the limitations imposed by the disease and its treatment. Similar results were found for individuals in a study who participated in a support group for patients with chronic respiratory problems.^5^ The authors concluded that a support group promotes the well-being, self-assurance and determination of each member, demonstrating that the repercussions of group discussions positively influenced the QoL of the study participants.^5^

The physical aspects, such as disease severity and motor complications, are considered important factors influencing the worsening of QoL of PD patients.^16,17^ This influence was confirmed in the present study, since there was a statistically significant correlation between worse motor capacity and low QoL of the subjects in the sample.

Besides motor symptoms, sedentary lifestyle and social isolation are also factors found to significantly impact perceived QoL in subjects with PD.^17^ The findings of the present study corroborated these observations, since subjects in the nPSG had higher levels of social phobia, isolating themselves from others as a consequence. This isolation results from the progressive characteristics of PD, which involve alterations in physical, mental and emotional aspects of the individual.^17^ Depression is a factor that strongly influences QoL in chronic neurological conditions. Previous studies have reported the association between depression and low QoL of patients with PD.^18,19^ In the present study, a correlation between depression scores and QoL was also found.

It has been suggested that depression, both in early- and late- onset PD, may be associated with impairments in activities of daily living.^20^ Studies have also suggested that depression is associated with motor function.^21-23^ Similarly, our results also suggested that depression is associated with motor impairment and lower performance in activities of daily living.

Depression and anxiety symptoms may precede motor symptoms by several years, suggesting that the neurobiological substrates of PD are responsible for these psychiatric disorders, at least in some of these patients.^24,25^ Although depression can result from the pathological process in PD, depressive symptoms can also stem from the emotional alterations following the discovery that they suffer from an incurable and progressive illness. Therefore, anxiety and its relationship with impaired QoL in PD patients may be understood from a psychological perspective, as a response to being diagnosed with a chronic, incurable and inexorable disease.^24^ Many patients will have difficulty coping with this new reality. Besides, social anxiety can also be explained by the difficulty in accepting the motor symptoms and alterations in appearance.

In the present study, depressive and anxiety symptoms were significantly milder in PSG subjects compared to those in the nPSG. It has been suggested that participating in a group with others having the same pathology makes patients feel more cheerful and helpful.^5^ Participation in PSG also creates the opportunity to make new friends. This helps the socializing process and probably has a positive influence on depressive and anxiety symptoms and social phobia. The relationship between lower levels of depression and greater social support was found in a cross-sectional study in a Chinese cohort.^26^

Our study has found that PD patients attending PSG showed better QoL, fewer depression and anxiety symptoms and less social phobia. However, it is not possible to definitively conclude whether these effects are a cause or a consequence of PSG attendance. These associations might be due to the fact that the presence of depressive and/or anxiety symptoms could promote higher levels of social phobia in these individuals who consequently experience more social isolation and have lower attendance in PSG.

In summary, the study showed that attending a support group for PD patients was positively associated with better QoL scores and fewer symptoms of depression, anxiety and social phobia.
